# The Efficacy and Safety of Polyethylene Glycol Cholesterol- and Tocopherol Polyethylene Glycol 1000 Succinate-Modified Transforming Growth Factor β1 Small Interfering RNA Lipid Nanoparticles in the Treatment of Paclitaxel-Resistant Non-Small-Cell Lung Cancer

**DOI:** 10.3390/pharmaceutics16010075

**Published:** 2024-01-04

**Authors:** Zhaowu Zeng, Xianglong Zeng, Xinyi Li, Yuxin Feng, Yue Kan, Xingyan Liu, Yiying Zeng

**Affiliations:** 1 School of Pharmacy, Hangzhou Normal University, Hangzhou 311121, China; 2 Guangdong Provincial Key Laboratory of Medical Molecular Diagnostics, Guangdong Medical University, Dongguan 523808, China

**Keywords:** PEG cholesterol, tocopherol polyethylene glycol succinate (TPGS), TGFβ1, siRNA, lipid nanoparticles, paclitaxel-resistant non-small-cell lung cancer

## Abstract

The aim of this study was to explore the efficacy and safety of TGFβ1 siRNA lipid nanoparticles (LNPs) modified with different PEG derivatives (PEG5000 cholesterol, abbreviated as CE; tocopherol polyethylene glycol 1000 succinate, abbreviated as TPGS) in the treatment of paclitaxel-resistant non-small-cell lung cancer. Three kinds of TGFβ1 siRNA LNPs were prepared via microfluidics technology, using different PEG derivatives and dosages (CE1.5, CE2.5, TPGS2.5) as variables. Their particle size, zeta potential, contents, and encapsulation efficiencies were determined. The inhibition of TGFβ1 mRNA and protein expression and the effects of the three kinds of LNPs on the proliferation of paclitaxel-resistant non-small-cell lung cancer cells (A549/T cell) were characterized. The distributions of the three siRNA LNPs in nude mice bearing A549/T tumors, especially at the tumor site, were observed using in vivo mouse imaging technology, and their corresponding efficacies were evaluated. The average particle size of the three kinds of TGFβ1 siRNA LNPs was about 70–80 nm, and they were capable of charge flipping. All three siRNA LNPs could effectively inhibit the expression of TGFβ1 mRNA and protein in A549/T cells and inhibit the proliferation of A549/T cells in vitro. The results of in vivo mice imaging showed that the three kinds of siRNA LNPs, when labeled with cypate, retain strong fluorescence in the tumor at 24 h. The pharmacodynamic results, such as for relative tumor volumes and tumor inhibition rates, reveal that TGFβ1 siRNA LNPs modified with CE1.5, CE2.5, or TPGS2.5 can be used to effectively treat paclitaxel-resistant lung adenocarcinoma. The histopathological results showed that the three kinds of LNPs have a certain toxicity but are relatively safe compared to common forms of chemotherapy such as cabazitaxel. TGFβ1 siRNA LNPs modified with CE1.5, CE2.5, and TPGS2.5 can inhibit TGFβ1 mRNA and protein expression in A549/T cells in vitro and can accumulate and play a role in the tumor tissue of nude mice, features that can be exploited for treating paclitaxel-resistant lung adenocarcinoma.

## 1. Introduction

Lung cancer is a common type of tumor. Taxanes such as paclitaxel and docetaxel are the first-line of drugs for lung cancer chemotherapy. However, with the wide application of taxanes, the drawback of taxane resistance becomes increasingly prominent, and this resistance is one of the main reasons for treatment failure [1]. Paclitaxel-resistant non-small-cell lung cancer is a common late-stage tumor in clinical practice, for which effective therapeutic drugs are lacking.

Transforming growth factor β1 (TGFβ1) is synthesized from a TGFβ-induced secretion protein, a multifunctional cell growth factor. TGFβ1 has growth inhibitory and anti-inflammatory roles during homeostasis and the early stages of cancer. Aberrant TGFβ activation in the late-stages of tumorigenesis, however, promotes the development of aggressive growth characteristics and metastatic spread [2]. TGFβ1 is hydrolyzed from the carboxyl terminal protein of a 390 amino acid precursor molecule [3], the gene for which is located in the 19q13.2 chromosome region, consisting of seven exons separated by six very large introns [4]. Moreover, silencing TGFβ1 expression in a tumor microenvironment can promote the differentiation of neutrophils into antitumor phenotypes [5,6]. Thus, TGFβ1 plays an important role in various advanced tumors.

Small interfering RNAs (siRNAs) may induce mRNA degradation and silence the gene encoding this mRNA [7,8]. Multiple siRNA formulations have been launched, such as Patisiran (Onpattro) in 2018. It was approved by the FDA for marketing in the treatment of the polyneuropathy of hereditary transthyretin-mediated amyloidosis [9]. siRNA can be used to treat tumors through an RNAi interference mechanism, and multiple formulations of this are currently undergoing clinical trials. As a therapeutic approach, RNAi can overcome the major drawbacks of traditional chemotherapy such as low tumor specificity, severe side effects, and the inability to inhibit undruggable targets such as transcription factors [10]. To effectively silence genes in the body, appropriate vectors are required for the delivery of siRNA. The ideal carrier delivery system should allow the avoidance of siRNA degradation by serum nuclease, the delivery of siRNA to target cells with high specificity and efficiency, the extension of the in vivo half-life, an improved cell uptake, the avoidance of MPS clearance, and have good biocompatibility, biodegradability, and non-immunogenicity [11]. The in vivo delivery of siRNA requires overcoming blood, tissue, cellular, and intracellular barriers in order to reach the target site and exert gene silencing. Blood stability, targeting, tumor permeability, and endosome escape ability are key properties of siRNA carriers [12]. Many nanocarrier systems have been developed for siRNA delivery to tumor tissues, and lipid nanoparticles (LNPs) remain one of the most attractive types of siRNA carriers [13]. 

LNPs have shown a great potential for delivering nucleic acid drugs [14]. An LNP is a type of vesicle with a lipid core, mainly composed of cholesterol, phospholipids, and polyethylene glycol (PEG)-conjugated lipids, in addition to ionizable cationic lipids [15,16]. Ionizable cationic lipids are essential for the efficient in vivo delivery of RNA by lipid nanoparticles (LNPs). Currently, DLin-MC3-DMA (abbreviated as MC3), ALC-0315, and SM-102 are the only ionizable cationic lipids clinically approved for RNA therapies. ALC-0315 and SM-102 are structurally similar lipids used in SARS-CoV-2 mRNA vaccines, while MC3 is used in siRNA therapy to knock down transthyretin in hepatocytes [17]. Dlin-MC3-DMA is a kind of ionizable cationic lipid that is the main excipient component of Onpattro. In circulation and at the physiological pH, ionizable cationic lipids adopt a net-neutral surface charge, avoiding the rapid clearance and toxicity associated with permanently cationic LNPs [18]. The PEG chain is located in the nanoparticle’s shell, which prevents the adsorption of serum proteins and uptake by the mononuclear phagocyte system, thereby prolonging the internal circulation time [19]. The main method for the preparation of siRNA LNPs is the microfluidics method, which allows control of the particle size and has a high siRNA encapsulation efficiency [20]. Based on the intermolecular interaction, the negatively charged nucleic acid and the positively charged lipid combine to form a nanostructure through an electrostatic interaction [21]. LNPs are usually associated with a low toxicity, a good complexability with siRNA, a high transfection efficiency, and good pharmacokinetic characteristics [22]. However, the safety of cationic lipids and PEG derivatives, which are the main lipid components in LNPs, still requires important consideration. The cationic lipids may stimulate the secretion of pro-inflammatory cytokines and reactive oxygen species and trigger inflammation, allergic reactions, immune reactions, and so on [23,24,25]. Due to the need for multiple applications in tumor treatment, the accumulation of these excipients and their impact on the immune system are of particular concern, which has resulted in efforts to substitute or modify these lipid components [26]. The safety of TGFβ1 siRNA also needs to be investigated: TGFβ1 siRNA was observed to have a relatively low toxicity in our previous studies [27], a point which will be further confirmed in this study.

LNP carriers can prevent siRNA from being degraded by serum nuclease and recognized by the immune system, and the specific LNP carrier determines the biological distribution of siRNA in the body. Adding PEG groups on the surface of an LNP can improve the stability of the siRNA [28], improve the biological activity, and reduce the interaction between the siRNA and the immune cells, nontarget tissues, and serum proteins. LNP is one of the most promising delivery carriers, but it is easily adsorbed by some specific lipoproteins, such as apolipoprotein E (ApoE), in the blood circulation and transported to the liver [29]. The problem to be solved when using LNP-based siRNA for cancer treatment is how to avoid LNP being adsorbed by lipoproteins so that a large amount of siRNA can reach the tumor site and achieve effective gene silencing. So far, several siRNA nanocarriers based on different materials have entered clinical trials for cancer treatment, but multiple clinical trials have been terminated due to efficacy or safety issues. At present, there are no officially launched antitumor drugs based on siRNA, and more in-depth research is needed on siRNA carrier delivery systems. In this study, we used TGFβ1 siRNA and modified LNPs with altered PEG derivatives (CE1.5 TGFβ1 siRNA LNP, CE2.5 TGFβ1 siRNA LNP, and TPGS2.5 TGFβ1 siRNA LNP; abbreviated as CE1.5 LNP, CE2.5 LNP, and TPGS2.5 LNP) to observe whether they can effectively accumulate in the tumor site, as well as their efficacy and safety on the treatment of paclitaxel-resistant lung adenocarcinoma, and we further explored the impact of these PEG derivatives on LNPs.

## 2. Materials and Methods

### 2.1. Materials

A microfluidics instrument (Inano, with SHM chip) was purchased from Shanghai micro-nano biologics Co., Ltd., Shanghai, China. The Spark microplate reader was from the Tecan company, Männedorf, Switzerland. The Nicomp Z3000 nanoparticle size potential analyzer was from Particle Sizing Systems (PSS), Billerica, MA, USA. The UV-2450 ultraviolet photometer was from the Shimadzu company, Kyoto, Japan. The Veriti 96-well thermal cycle type ordinary gradient PCR instrument was from the ABI company, Tampa, FL, USA. The fluorescence quantitative PCR circulator was from ABI, USA. The electrophoresis apparatus was from Bio-rad, Hercules, CA, USA. The Trans blot turbo versatile protein transfer system was from Bio-rad, USA. The ChemiDoc touch imaging system was from Bio-rad, USA. The XD-101 CO_2_ incubator was from the Sanyo company, Tokyo, Japan. The IX51 biological inverted microscope was from Olympus, Tokyo, Japan. 

The TGFβ1 siRNA (sense (5′-3′): AACGAAAUCUAUGACAAGUUC); antisense (5′-3′): ACUUGUCAUAGAUUUCGUUGU) was from Bioengineering (Shanghai, China) Co., Ltd., China. The Dlin-MC3-DMA, DSPC (1,2-distearoyl-sn-glycero-3-phosphocholine), and cholesterol were purchased from the AVT (Shanghai, China) pharmaceutical technology Co., Ltd., China. The PEG5000 cholesterol (CE) was from Shanghai ponsure biotechnology Co., Ltd., Shanghai, China. The TPGS was from the Shanghai Changwei pharmaceutical accessories technology Co., Ltd., Shanghai, China. The acetic acid sodium acetate buffer (Rnase-free, pH 4.0) was from the Leagene corporation, Beijing, China. The ultrafiltration centrifuge tubes (100 KD) were from the Shanghai Pall company, China. The solution of 100 mM PB was from Shanghai dingguo biotechnology Co., Ltd., Shanghai, China. The Diethypyrocarbonate (DEPC)-treated water was from EMD millicore Co., Shanghai, China. The Quant-it Ribogreen RNA reagent was from Thermo fisher Co., Shanghai, China. The Emulsifier OP was from the China national pharmaceutical group chemical reagent Co., Ltd. The TRIzol was from the Invitrogen company, Waltham, MA, USA. The cDNA first-strand synthesis kit and TB green premix ex Taq II (Tli RnaseH Plus) were from TaKaRa, Kyoto, Japan. The TGFβ1 primers (forward: CAGCAACAATTCCTGGCGATA; reverse: AGTGTGTTATCCCTGCTGTCA) were from Jiangsu keygen biotechnology Co., Ltd., Nanjing, China. The β-action primers (forward: CCACGAACTACCTTCAACTCC; reverse: CTTGATCTTCATTGTGCTGGGGT) were from Jiangsu keygen biotechnology Co., Ltd., China. The rabbit anti-β-actin (molecular weight 42 kDa) and rabbit anti-TGFβ1 (molecular weight 44 kDa) were from Abcam, Cambridge, UK. The sheep anti-rabbit IgG HRP, whole protein extraction kit, BCA protein content detection kit, SDS-PAGE gel preparation kit, 5 × SDS-PAGE protein loading buffer, 1 × Tris glycine protein electrophoresis buffer, Western blotting detection kit, developing and fixing reagent, trypsin EDTA digestion solution, RPMI-1640, and CCK8 kits were all purchased from Jiangsu keygen biotechnology Co., Ltd., China. The fetal bovine serum was from the Gibco corporation, Shanghai, China. The rabbit anti-TGFβ1 (dilution ratio 1:200) was from the Abcam company, China. The MaxVision reagent kit (rabbit) was from Meixin biotechnology Co., Ltd., Fuzhou, China. The Cypate was from Hangzhou xinqiao biotechnology Co., Ltd., Hangzhou, China.

The A549/taxol (A549/T, A549 cell with taxol resistance) cell line was provided by Jiangsu keygen biotechnology Co., Ltd., China. The complete culture medium was 90% of RPMI-1640 combined with 10% of FBS and was incubated in a 37 °C, 5% CO_2_, and saturated humidity incubator. BALB/c nude mice were purchased from Shanghai lingchang biotechnology Co., Ltd., Shanghai, China.

### 2.2. Preparation of TGFβ1 siRNA LNPs

The formulation for CE1.5 (i.e., the molar ratio of CE in the lipid composition was 1.5%) was MC3/DSPC/cholesterol/PEG5000-CE with a molar ratio of 45/10/43.5/1.5. The formulation for CE2.5 (i.e., the molar ratio of CE was 2.5%) was MC3/DSPC/cholesterol/PEG5000-CE with a molar ratio of 45/10/42.5/2.5. The formulation for TPGS2.5 (i.e., the molar ratio of TPGS was 2.5%) was MC3/DSPC/cholesterol/TPGS with a molar ratio of 45/10/42.5/2.5. The formulation for the NC siRNA LNP (negative control siRNA LNP) and blank LNP (it does not have siRNA) was in line with the formulation of the CE2.5 LNP. The preparation method for the siRNA LNP was as follows: 1.5 mL of organic phase solution was prepared according to the prescription, and 1 mg of siRNA was dissolved in 4.5 mL of acetic acid sodium buffer to form an aqueous phase. A microfluidics instrument was used to prepare samples with a 1:3 ratio of organic phase to aqueous phase and a total flow rate of 12 mL/min, and an ultrafiltration centrifuge tube was then used to remove the free siRNA, sodium acetate buffer, and ethanol. The volume was adjusted using a phosphate buffer, and the final sample was then obtained following sterilization and filtration using a 0.22 μm disposable filter.

### 2.3. Particle Morphology, Size Distribution, and Zeta Potential of TGFβ1 siRNA LNPs

The particle sizes and size distributions of the siRNA LNPs were detected using a nanoparticle potential analyzer (PSS company, Perm, Russia) by diluting an appropriate amount of sample to the appropriate concentration. The parallel detection was performed three times. The zeta potential of the siRNA LNPs was detected using a nanoparticle potential analyzer. An appropriate amount of sample was taken and diluted to the appropriate concentration using either a pH 4 sodium acetate buffer or a pH 7.4 phosphate-buffered solution, and parallel detection was performed three times.

### 2.4. Detection of the Contents and Encapsulation Efficiencies of siRNA in TGFβ1 siRNA LNPs

The contents and encapsulation efficiencies of the siRNA in the TGFβ1 siRNA LNPs were detected using the RiboGreen method and the ultrafiltration centrifugation method, respectively. A detailed description of these methods can be found in the Supplementary Materials Figures S1 and S2.

### 2.5. Inhibition of TGFβ1 mRNA Expression in A549/T Cell after siRNA LNP Intervention

A549/T cells were inoculated in a six-well plate culture dish corresponding to a density of 1 × 10^5^/well and incubated for 24 h. The drugs were added until the cells reached about a 60% confluency. The drug concentration of the siRNA LNP was 4 μg/mL, and culturing was carried out for 48 h. Then, the mRNA expression in these A549/T cells was detected according to the common qPCR method, and a detailed description of this method can be found in the Supplementary data.

### 2.6. Inhibition of TGFβ1 Protein Expression in A549/T Cell after siRNA LNP Intervention

A549/T cells were inoculated into a six-well plate at a density of 1 × 10^5^/mL and incubated at 37 °C and 5% of CO_2_ for 24 h. After 24 h, the solution was changed to 2 mL of culture medium containing the corresponding drug, with siRNA concentrations of 4 μg/mL. After 72 h of drug intervention, the culture medium was discarded, washed twice with pre-cooled PBS. The PBS was discarded, and the protein was then extracted. Protein quantification was performed using the BCA method, followed by SDS-PAGE electrophoresis and a membrane transfer. Primary antibody rabbit anti-TGFβ1 and secondary antibody sheep anti-rabbit IgG-HRP were used for immunoblotting; the ECL chemiluminescence kit was used for color development; GBOX chemiXR5 imaging was used, and the results were subjected to a grayscale analysis using the Gel-Po32 software.

### 2.7. In Vitro Proliferation Inhibition of siRNA LNP in A549/T Cells

A549/T cells were digested and counted; a cell suspension with 3.5 × 10^4^ cells/mL was prepared, and 100 μL of cell suspension per well was added into a 96-well cell culture plate. The cells were then incubated at 37 °C, with 5% of CO_2_ for 24 h. The drugs were diluted to the working solution concentration with the culture medium, and 100 μL of culture medium containing the corresponding drug was added to each well. A negative control group was also established. The 96-well cell culture plate was incubated for 72 h. Then, 10 μL of CCK-8 solution was added to each hole, incubation continued for 2–3 h, followed by gentle mixing in a shaker for 10 min, and bubbles were then removed from the 96-well plate. A microplate reader was used to read the OD value of each well (λ = 450 nm), and the inhibition rate was calculated. The inhibition rate (%) = (OD value of the negative control group—OD value of the experimental group)/OD value of the negative control group × 100%.

### 2.8. Tissue Distributions of siRNA LNPs in Tumor-Bearing Nude Mice Observed Using an In Vivo Mice Imaging System

Nude mice bearing tumors were randomly divided into three groups, with five mice in each group, administered three cypate-labeled LNPs at a dose of 0.25 mg/kg of cypate via the tail vein. They were labeled with cypate for the CE1.5 LNP, CE2.5 LNP, and TPGS2.5 LNP groups. After injecting a sample containing the same amount of cypate through the tail vein, fluorescence distributions were observed using a mice imaging system at 0, 0.25, 0.5, 1, 2, 4, 21, and 24 h after anesthesia administration. The excitation wavelength was 745 nm, the emission wavelength 820 nm, and the fluorescence of the tumor was quantified at different time points. The final average fluorescence intensity at each time point was the measured average fluorescence intensity at each time point minus the average fluorescence intensity at 0 h, thereby reducing the impact of the fluorescence value of the nude mouse’s tumor itself on the results. The average fluorescence intensity–time curve of the tumor was drawn. The statistical moment model of the DAS32.8 software (BioGuide Co., Shanghai, China) was used to calculate the dynamic parameters of fluorescence intensity in the tumor tissue. The SPSS 18.0 software ANOVA method was used for a statistical analysis. *p* < 0.05 indicated statistically significant differences, while ns, *p* > 0.05, indicated no statistically significant differences.

### 2.9. Pharmacodynamic Study of siRNA LNPs in Tumor-Bearing Nude Mice

Cultured A549/T suspension cells at a concentration of 5 × 10^7^/mL were collected and subcutaneously inoculated in the right armpit of 4-week-old female BALB/c nude mice at a volume of 0.1 mL per animal. When the tumor had grown to about 100–150 mm^3^, the animals were randomly divided into four groups, with seven mice in each group. The dosages were as follows: a solvent group (0.9% sodium chloride injection) of 0.2 mL; CE1.5, CE2.5, and TPGS2.5 TGFβ1 siRNA LNP groups, all injected with 1 mg/kg. The tumor volume was recorded every two days. The formula for calculating tumor volume (TV) is TV = 0.5 × a × b^2^, where a and b represent the length and width, respectively. The relative tumor volume (RTV) was calculated based on the measurement results using the formula RTV = V_t_/V_0_, where V_0_ is the tumor volume measured during cage administration, and V_t_ is the tumor volume at each measurement. The tumor inhibition rate (%) was calculated as follows: tumor inhibition rate (%) = (average tumor weight of the control group—average tumor weight of the treatment group)/average tumor weight of the control group×100%. After treatment, the tumor tissue was taken out and we detected the TGFβ1 protein expression in the tumor tissue according to conventional immunohistochemical methods. The optical density value of TGFβ1 staining was calculated using the ImageJ software 1.53e (National Institutes of Health, USA). The SPSS 18.0 software ANOVA method was used for a statistical analysis. A statistical difference was defined as significant at * *p* < 0.05, ** *p* < 0.01, *** *p* < 0.001, and **** *p* < 0.0001, while not significant (ns) at *p* > 0.05.

## 3. Results and Discussion

### 3.1. The Results of Particle Morphology, Size Distribution, and Zeta Potential of TGFβ1 siRNA LNPs

**Figure 1:**
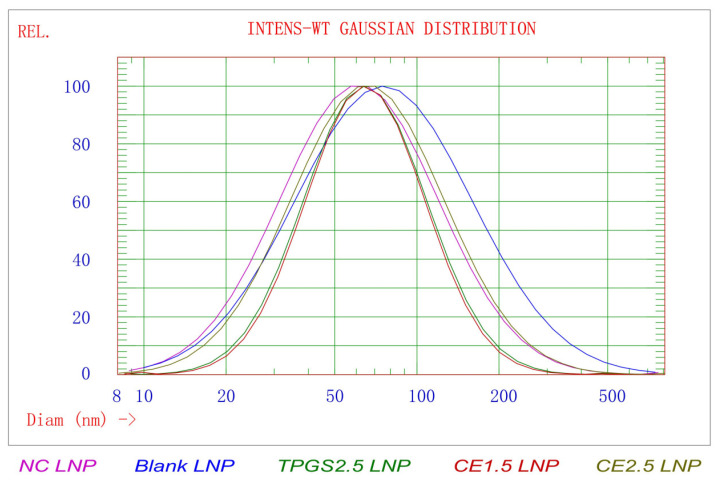
The particle size and distribution of the LNPs. The average particle size of the prepared LNP is about 70–90 nm. The particle size and PDI of the CE2.5 LNP has a slightly larger particle size and a larger PDI, while the others were similar.

The particle size results showed that the average particle size of the prepared siRNA LNP is about 70–90 nm, as shown in Figure 1 and Table 1. The particle size and PDI of each prescription differed slightly, with the CE2.5 LNP having a slightly larger particle size and a larger PDI, while the others were similar. The amount of CE appears to affect the particle size and PDI, with a special impact on the PDI. The zeta potential results showed that the zeta potential in the pH4 buffer followed the order TPGS2.5 LNP > CE1.5 LNP > CE2.5 LNP, and the zeta potential in the pH 7.4 buffer was similar. The three preparations were negatively charged in the pH7.4 buffer solution and positively charged in the pH4 buffer solution, exhibiting a charge reversal function. This indicates that they are electronegative in blood and would not be easily adsorbed, but they become positively charged in the endosome, thus facilitating the function of endosome escape.

There are reports suggesting that the proportions of different lipid species in optimized LNP-siRNA systems may vary according to the particular ionizable cationic lipid employed [30]. The average particle sizes of the CE1.5 and CE2.5 LNPs were similar, but the increased PDI indicates that the increase in CE dosage leads to a decreased uniformity of the particle size. The average particle sizes and PDI of the TPGS2.5 and CE1.5 LNPs were similar, indicating that different PEG derivatives can be used to prepare LNPs with similar size and size distributions. However, the zeta potential of the TPGS2.5 LNP was higher than that of the CE1.5 and CE2.5 LNPs. The molecular weight of the PEG in the TPGS was about 1000, while the molecular weight of the PEG in the PEG5000 cholesterol was about 5000. Meanwhile, the zeta potential of the CE2.5 LNP was slightly smaller than that of the CE1.5 LNP. Therefore, the molecular weight and dosage of PEG in PEG derivatives can be adjusted to obtain LNPs with appropriate potential values for adapting to their different applications.

### 3.2. The Results of siRNA Contents and Encapsulation Efficiencies in siRNA LNPs

The emission wavelength was 530 nm. The excitation wavelength was 485 nm. The standard curve equation for TGFβ1 siRNA is y = 82.174*x* + 598.45, with *r* = 0.9998, as shown in Figure S3. The concentration of the CE1.5 LNP was (86.03 ± 1.66) μg/mL. The concentration of the CE2.5 LNP was (90.69 ± 1.58) μg/mL. The concentration of the TPGS2.5 LNP was (87.03 ± 1.90) μg/mL. The encapsulation efficiency of the CE1.5 LNP was 94.79% ± 2.50%, that of the CE2.5 LNP was 94.42% ± 3.55%, and that of the TPGS2.5 LNP was 102.50% ± 3.56%. The contents and encapsulation efficiencies of the TPGS2.5, CE1.5, and CE2.5 LNPs were similar, indicating that the amount of encapsulated siRNA may be more closely related to the type and dosage of other lipids, especially cationic lipids.

### 3.3. TGFβ1 mRNA Expression Inhibition by the Three siRNA LNPs

**Figure 2:**
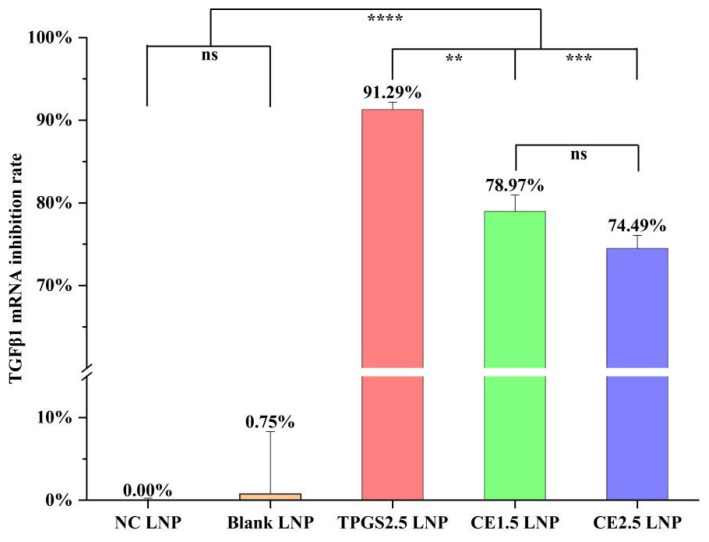
The inhibition rates of TGFβ1 mRNA expression of the various siRNA LNPs (*n* = 4. ns, *p* > 0.05; ** *p* < 0.01; *** *p* < 0.001; and **** *p* < 0.0001). Compared to the other three groups, the differences with the TGFβ1 mRNA inhibition rates for the NC LNP and blank LNP were statistically significant, *p* < 0.0001. These results indicate that TPGS2.5, CE1.5, and CE2.5 LNPs can effectively inhibit TGFβ1 mRNA expression in A549/T cells.

The rate of TGFβ1 mRNA expression inhibition in the A549/T cells was found to be 0.00% ± 0.24% for the NC LNP, 0.75% ± 7.54% for the blank LNP, 91.29% ± 0.90% for the TPGS2.5 LNP, 78.97% ± 1.99% for the CE1.5 LNP, and 74.49% ± 1.59% for the CE2.5 LNP, as shown in Figure 2. The results showed that the rate of TGFβ1 mRNA expression inhibition followed the order TPGS2.5 LNP > CE1.5 LNP > CE2.5 LNP. Through a statistical analysis using the SPSS18.0 ANOVA (Sidak) method, it was found that there was no statistically significant difference in the rates of TGFβ1 mRNA expression inhibition of the NC and blank LNPs, *p* > 0.05. Compared to the other three groups, the differences with the TGFβ1 mRNA inhibition rates for the NC LNP and blank LNP were statistically significant, *p* < 0.0001. There was no statistically significant difference in the rates of TGFβ1 mRNA expression inhibition for the CE1.5 and CE2.5 LNPs, *p* > 0.05. There was a statistically significant difference in the rate of TGFβ1 mRNA inhibition between the TPGS2.5 LNP and the CE1.5 LNP, *p* < 0.01, as well as between the TPGS2.5 LNP and the CE2.5 LNP, *p* < 0.001. These results indicate that TPGS2.5, CE1.5, and CE2.5 LNPs can effectively inhibit TGFβ1 mRNA expression in A549/T cells.

### 3.4. TGFβ1 Protein Expression Inhibition by the Three siRNA LNPs

**Figure 3:**
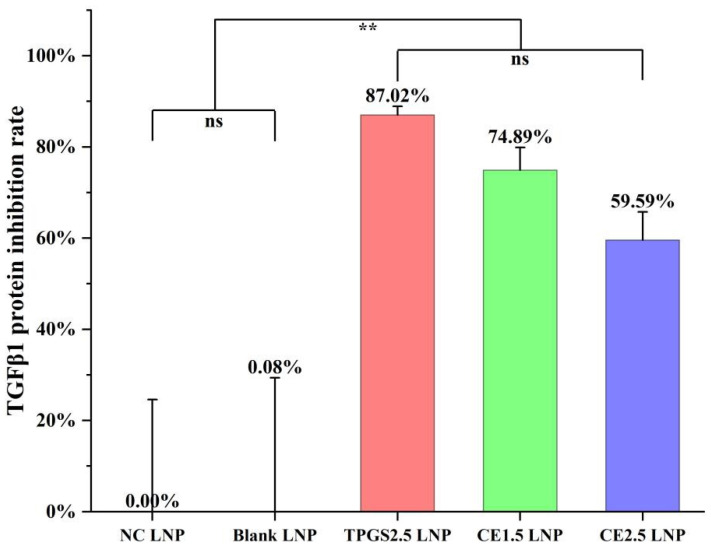
The inhibition rates of TGFβ1 protein expression of the various siRNA LNPs (*n* = 4. ns, *p* > 0.05; ** *p* < 0.01). Compared to the NC siRNA and blank LNP groups, the other three groups show statistically significant differences in the rates of TGFβ1 protein inhibition, *p* < 0.01, indicating that all those three formulations can effectively inhibit TGFβ1 protein expression.

The rate of TGFβ1 protein expression inhibition in the A549/T cells was found to be 0.00% ± 24.60% for the NC siRNA LNP, 0.08% ± 29.29% for the blank LNP, 87.02% ± 1.89% for the TPGS2.5 LNP, 74.89% ± 5.00% for the CE1.5 LNP, and 59.59% ± 6.16% for the CE2.5 LNP, as shown in Figure 3 and Figure S4. The results showed that the rate of TGFβ1 protein expression inhibition followed the order TPGS2.5 LNP > CE1.5 LNP > CE2.5 LNP. A statistical analysis using the SPSS 18.0 ANOVA (Sidak) method revealed that there was no statistically significant difference in the TGFβ1 protein inhibition rates of the NC siRNA and blank LNPs, *p* > 0.05. Compared to the NC siRNA and blank LNP groups, the other three groups showed statistically significant differences in the rates of TGFβ1 protein inhibition, *p* < 0.01. There were no statistically significant differences in the protein inhibition rates among the three formulations of TPGS2.5, CE1.5, and CE2.5 LNPs, *p* > 0.05, indicating that all those three formulations could effectively inhibit TGFβ1 protein expression. There is a certain correlation between the in vitro protein expression inhibition and the dosage and molecular weight of the PEG derivative.

### 3.5. The In Vitro A549/T Cell Proliferation Inhibition for siRNA LNPs 

The inhibition of A549/T cell proliferation for the different siRNA LNPs is shown in Figure S5. The IC_50_ on A549/T cells was 47.59 μg/mL for the CE1.5 LNP, 43.64 μg/mL for the CE2.5 LNP, 54.93 μg/mL for the TPGS2.5 LNP, and 82.92 μg/mL for the NC LNP. The results show that CE1.5 LNP, CE2.5 LNP, and TPGS2.5 LNP have similar inhibition effects on A549/T cells. The NC siRNA LNP also had a certain inhibitory effect, which we speculate to be caused by excipient toxicity. The three kinds of LNPs had similar cell inhibition rates, which is not inconsistent with the results for the rates of mRNA and protein expression inhibition.

### 3.6. The Tissue Distribution of siRNA LNP in Tumor-Bearing Nude Mice Determined Using an In Vivo Mice Imaging System

#### 3.6.1. Tissue Distribution Imaging of Cypate-Labeled siRNA LNPs in Tumor Tissue

**Figure 4:**
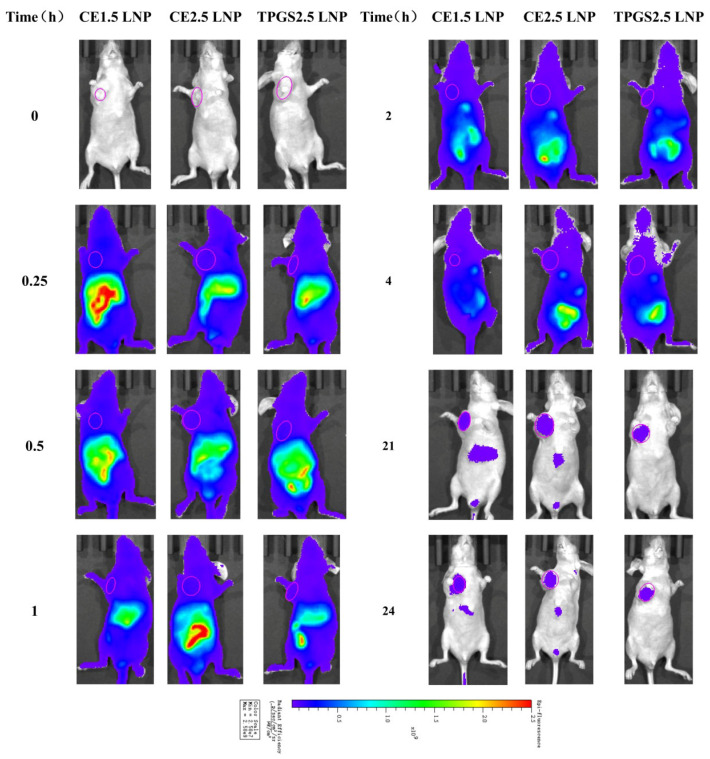
In vivo mice imaging of the three siRNA LNPs labeled with cypate. The SiRNA LNPs labeled with cypate fluorescence were rapidly distributed throughout the body, with the strongest fluorescence in abdominal organs. The tumor site still retained a strong fluorescence at 24 h.

The tissue distribution of the three siRNA LNPs in mice at different times was detected. It was found that the siRNA LNPs labeled with cypate fluorescence of different formulations were rapidly distributed throughout the body after tail vein administration, with the strongest fluorescence in abdominal organs. With the extension of time, especially at 24 h, the overall fluorescence basically disappeared, while the tumor site still retained a strong fluorescence. Of the studied formulations, the fluorescence intensity of the CE1.5 and CE2.5 LNPs was slightly stronger than that of the TPGS2.5 from 0.25 to 1 h, and then, subsequently, similar in the three groups, as shown in Figure 4.

#### 3.6.2. The Fluorescence Intensity Dynamics of Cypate-Labeled siRNA LNPs in Tumor Tissue

The fluorescence intensity–time curves of the cypate-labeled siRNA LNPs in the tumor are shown in Figure S6. The fluorescence intensity gradually decreases with time. After a natural logarithm conversion of the two main fluorescence intensity kinetic parameters AUC(0–t) and Cmax of the three cypate-labeled siRNA LNPs, a statistical analysis was carried out using the SPSS 18.0 ANOVA (Sidak) test. The results show that there is no statistically significant difference in AUC(0–t) and Cmax between CE1.5 LNP, CE2.5 LNP, and TPGS2.5 LNP, *p* > 0.05. Their kinetic parameters in the tumors were similar, as shown in Table 2. The cumulants of the PEG-modified LNPs with different molecular weights and dosages in the tumor were similar, which may be due to the influence of various components such as lipoproteins in the blood on the three types of LNPs, suggesting that blood circulation has a very important impact on the distribution of the LNPs.

### 3.7. The Results of Efficacy, Tumor Immunohistochemistry, and Organ Pathology of the Three siRNA LNPs for the Treatment of Paclitaxel-Resistant Lung Adenocarcinoma

#### 3.7.1. The Efficacies of the Three siRNA LNPs in the Treatment of Paclitaxel-Resistant Lung Adenocarcinoma

**Figure 5:**
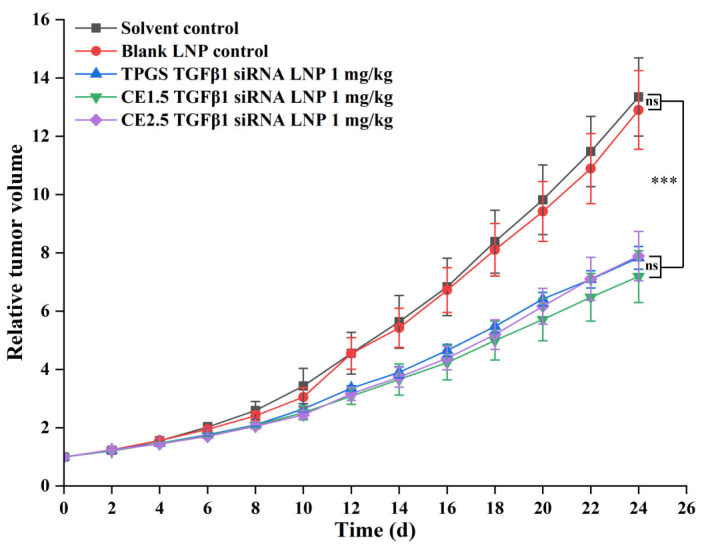
The relative tumor volume of the three siRNA LNPs (*n* = 7. Ns, *p* > 0.05; *** *p* < 0.001). These groups (CE1.5 LNP, CE2.5 LNP, and TPGS2.5 LNP groups) are statistically significantly different compared to the solvent control group and the blank LNP control group (*p* < 0.001), indicating that they can effectively inhibit paclitaxel-resistant lung adenocarcinoma.

The relative tumor volumes are shown in Figure 5, and they were statistically analyzed using the SPSS18.0 ANOVA (LSD) method. The results show that there is no statistically significant difference in the tumor inhibition rates between the CE1.5 LNP, CE2.5 LNP, and TPGS2.5 LNP groups, but that these groups are statistically significantly different compared to the solvent control group and the blank LNP control group (*p* < 0.001).

**Figure 6:**
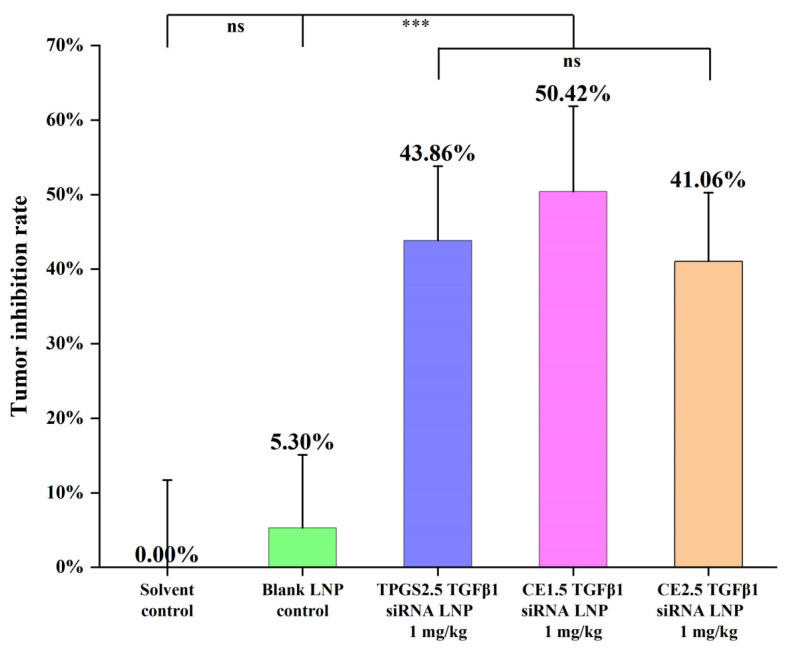
Tumor inhibition rates of the three siRNA LNPs (*n* = 7. ns, *p* > 0.05; *** *p* < 0.001). These groups (CE1.5 LNP, CE2.5 LNP, and TPGS2.5 LNP groups) are statistically significantly different compared to the solvent control group and the blank LNP control group (*p* < 0.001), indicating that they can effectively inhibit paclitaxel-resistant lung adenocarcinoma.

**Figure 7:**
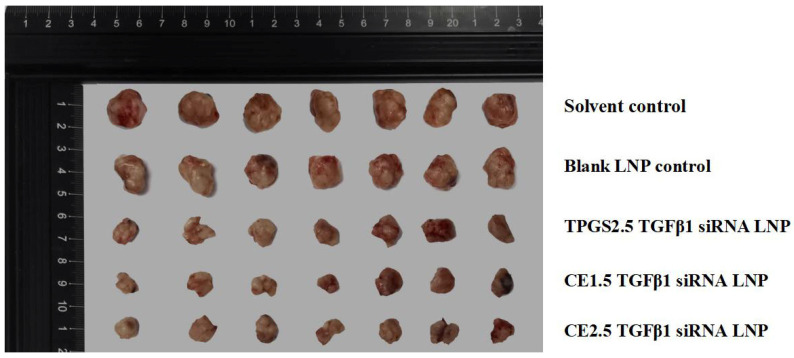
Tumor tissues of the various groups.

After the administration cycle, the tumor was removed and weighed, and the tumor inhibition rates are shown in Figure 6. The tumor tissues of the various groups are shown in Figure 7. The tumor inhibition rate was 43.86% ± 9.97% for the TPGS2.5 LNP, 50.42% ± 11.44% for the CE1.5 LNP, and 41.06% ± 9.21% for the CE2.5 LNP. The efficacy results showed that the tumor inhibition rate followed the order CE1.5 LNP > TPGS2.5 LNP ≈ CE2.5 LNP. A statistical analysis was conducted using the SPSS18.0 ANOVA (LSD) method. The results show that there is no statistically significant difference in the tumor inhibition rates between the CE1.5 LNP, CE2.5 LNP, and TPGS2.5 LNP groups. There is a statistically significant difference for the CE1.5 LNP, CE2.5 LNP, or TPGS2.5 LNP groups compared to the solvent control group, while the tumor inhibition rates are significantly higher than for the control group (*p* < 0.001). The efficacy results suggested that a PEG of different dosages or molecular weights produces similar therapeutic effects when comparing CE1.5 LNP, CE2.5 LNP, and TPGS2.5 LNP. Compared to the mRNA and protein inhibition results mentioned above, this suggests that the in vitro mRNA and protein effect in cells may partially differ from the proliferation inhibition rates in vitro in tumor cells, or in vivo in animals. This may be related to differences in the distribution and metabolism of siRNA LNPs. 

The changes in body weight of the nude mice during the administration cycle are shown in Figure S7. For the statistical analysis, the SPSS18.0 ANOVA (Sidak) method was used. It was found that the weight of all four groups of nude mice did not decrease. There was no statistically significant difference in the body weight between the four groups at the end of the experiment, *p* > 0.05. This indicates that the three siRNA LNPs are relatively safe.

#### 3.7.2. The Tumor Immunohistochemistry Results of the Three siRNA LNPs after the Treatment of Paclitaxel-Resistant Lung Adenocarcinoma

**Figure 8:**
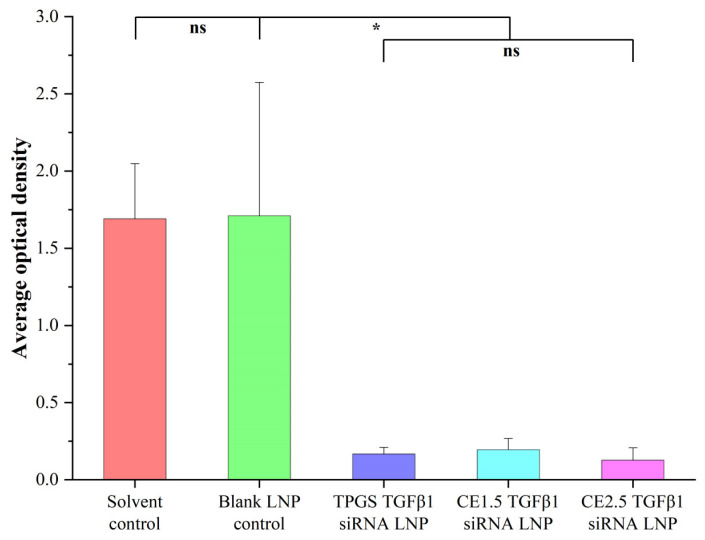
TGFβ1 protein expression in tumor tissue after administration (*n* = 6, ns, *p* > 0.05; * *p* < 0.05). TGFβ1 protein expression in the CE1.5 LNP, CE2.5 LNP, and TPGS2.5 LNP groups was significantly reduced compared to the two control groups, indicating their good inhibition effects on the TGFβ1 protein.

The tumor immunohistochemistry results for the three siRNA LNPs after the treatment of paclitaxel-resistant lung adenocarcinoma are shown in Figure 8 and Figure S8. A statistical analysis was conducted using the SPSS 18.0 ANOVA (Games-Howell) method. The difference of TGFβ1 protein expression between the solvent control group and the three siRNA LNPs groups (CE1.5 LNP, CE2.5 LNP, and TPGS2.5 LNP groups) was statistically significant, *p* < 0.001. TGFβ1 protein expression in the CE1.5 LNP, CE2.5 LNP, and TPGS2.5 LNP groups was significantly reduced compared to the blank LNP control group (*p* < 0.05). The difference in TGFβ1 protein expression between the CE1.5 LNP, CE2.5 LNP, and TPGS2.5 LNP groups was not statistically significant, *p* > 0.05.

#### 3.7.3. The Organ Pathology Results after the In Vivo Treatment of Paclitaxel-Resistant Lung Adenocarcinoma

The pathological detailed results of various organs of the five groups after the efficacy experiment are shown in the Supplementary data and Figure S9. The histopathological results after multiple doses showed that the TPGS2.5 LNP, the CE1.5 LNP, and the CE2.5 LNP had a significant effect on destroying the tumor cells. The CE1.5 LNP and the CE2.5 LNP had little impact on the heart, while the CE2.5 LNP had a slight impact on the heart. The CE1.5 LNP, CE2.5 LNP, and TPGS2.5 LNP had similar effects on the liver. The three types of LNP had no significant effects on the spleen. The TGFβ1 siRNA and LNP excipients had a relatively low toxicity. In a word, the three types of LNP had a certain toxicity mainly related to inflammation in the lungs, liver, kidneys, and stomach. Compared to chemotherapeutics such as cabazitaxel in our previous work, cabazitaxel showed greater cellular and in vivo toxicity than the siRNA LNP [27]. In this study, the toxic and side effects of TGFβ1 siRNA LNPs modified with CE1.5, CE2.5, and TPGS2.5 are relatively slight, suggesting that the three kinds of TGFβ1 siRNA LNP are relatively safe.

Lipid nanoparticle (LNP) systems are composed of ionizable cationic lipids, phospholipid, cholesterol, and polyethylene glycol (PEG)-lipids and are produced through the rapid mixing of an ethanolic-lipid solution with an acidic aqueous solution, followed by dialysis into a neutralizing buffer [31]. siRNA-based gene silencing is crucial for those targets that are not druggable or accessible to small molecules, antibodies, or proteins. Moreover, siRNA has shown great promise in potentiating chemotherapy by sensitizing drug-resistant cancer cells [32]. In our previous report, we found that TGFβ1 siRNA lipid nanoparticles modified with PEG2000-c-DMG could inhibit paclitaxel-resistant non-small-cell lung cancer [27]. In this study, we used TGFβ1 siRNA LNPs modified with CE1.5, CE2.5, and TPGS2.5 to treat paclitaxel-resistant non-small-cell lung cancer and found that the effect was similar to when we used LNPs modified with PEG2000-c-DMG in our previous report [27]. This indicates that PEG derivatives of different molecular weight and dosage can be used to modify TGFβ1 siRNA LNPs in treating paclitaxel-resistant non-small-cell lung cancer in vitro and in vivo. According to a report, PEG-DSPE in a lipid nanoparticle formulation (LNPK15) is more rapidly degraded than siRNA and other lipids in both mice and monkeys. LNPK15 acquires an increased knockdown in activity after undergoing PEG-DSPE hydrolysis in vivo, which is a key mechanism for achieving both a long circulation and a potent knockdown efficiency [33]. Our research results indicate that these PEG derivatives exhibited certain differences in their in vitro effects, but that their in vivo efficacies were similar, although the molecular weight and dosage of the PEG derivatives varied. This may be related to the hydrolysis rate of the PEG derivatives in blood circulation and tissues. In short, the results of this study suggest that appropriate PEG derivatives could be used to prepare TGFβ1 siRNA LNPs, such as CE1.5 LNP, CE2.5 LNP, and TPGS2.5 LNP, for successfully treating paclitaxel-resistant lung cancer in nude mice and that they are relatively safe. However, the efficacy and safety of these siRNA LNPs still needs improvement, and the development of active tumor targeting and better ionizable cationic lipids represents a possible main research direction.

## 4. Conclusions

PEG cholesterol- and TPGS-modified LNPs can effectively load TGFβ1 siRNA. TGFβ1 siRNA LNPs modified with CE1.5, CE2.5, and TPGS2.5 can efficiently inhibit TGFβ1 mRNA and protein expression in A549/T cells in vitro, and they can accumulate and play a role in the tumor tissue of nude mice. TGFβ1 siRNA LNPs modified with CE1.5, CE2.5, or TPGS2.5 could be used to effectively treat paclitaxel-resistant lung adenocarcinoma. They had a certain toxicity but were relatively safe compared to common forms of chemotherapy such as cabazitaxel and, thus, have the potential to be used for treating paclitaxel-resistant lung adenocarcinoma. More in-depth research is required to develop more effective, safer siRNA LNP systems.

## Figures and Tables

**Table 1:** The results of the particle size, distribution, and zeta potential of the LNPs (*n* = 3).

LNPs	Size (nm)	STD * (nm)	PDI **	90% of Distribution < (nm)	Zeta Potential in pH 4 Buffer (mV)	Zeta Potential in pH 7.4 Buffer (mV)
CE1.5 LNP	74.0 ± 1.6	37.6 ± 2.1	0.259 ± 0.018	132.9 ± 5.3	11.71 ± 0.58	−6.27 ± 0.54
CE2.5 LNP	81.9 ± 1.9	53.2 ± 3.2	0.422 ± 0.032	169.1 ± 7.9	7.73 ± 0.91	−8.76 ± 0.39
TPGS2.5 LNP	73.9 ± 0.5	38.4 ± 0.6	0.270 ± 0.005	134.3 ± 1.5	45.06 ± 2.05	−5.11 ± 0.63
NC LNP	77.1 ± 1.1	50.3 ± 0.6	0.426 ± 0.020	159.9 ± 0.9	5.71 ± 0.23	−9.91 ± 0.73
Blank LNP	98.5 ± 7.2	72.7 ± 6.4	0.544 ± 0.018	221.1 ± 18.3	8.09 ± 0.89	−6.32 ± 0.31

* STD, standard deviation. ** PDI, polydispersity index.

**Table 2:** The dynamic parameters for fluorescence intensity of cypate-labeled siRNA LNPs in tumor tissue.

Parameter	Units	CE1.5 LNP		CE2.5 LNP		TPGS2.5 LNP	
		Mean	SD	Mean	SD	Mean	SD
AUC(0–t)	AFE × h	1.42 × 10^9^	3.04 × 10^8^	1.39 × 10^9^	1.20 × 10^8^	1.22 × 10^9^	1.44 × 10^8^
AUC(0–∞)	AFE × h	2.33 × 10^9^	1.33 × 10^9^	1.69 × 10^9^	1.76 × 10^8^	1.48 × 10^9^	1.74 × 10^8^
R_AUC(t/∞)	%	69.68	19.671	82.7	7.276	82.82	6.687
AUMC(0–t)	h × h × AFE	1.11 × 10^10^	3.93 × 10^9^	8.86 × 10^9^	5.78 × 10^8^	8.04 × 10^9^	7.99 × 10^8^
AUMC(0–∞)	h × h × AFE	7.16 × 10^10^	1.01 × 10^11^	2.07 × 10^10^	6.60 × 10^9^	1.84 × 10^10^	6.44 × 10^9^
MRT(0–t)	h	7.66	1.28	6.395	0.657	6.604	0.724
MRT(0–∞)	h	22.703	18.142	12.093	3.282	12.308	3.553
VRT(0–t)	h × h	62.863	11.637	49.612	6.999	52.792	8.885
VRT(0–∞)	h × h	896.19	1316.742	233.055	107.471	241.478	129.526
λz	1/h	0.055	0.026	0.073	0.017	0.071	0.015
C_last	AFE	2.90 × 10^7^	1.43 × 10^7^	2.00 × 10^7^	5.75 × 10^6^	1.70 × 10^7^	3.36 × 10^6^
t1/2z	h	17.001	12.691	9.917	2.327	10.218	2.709
Tmax	h	0.9	0.224	0.8	0.274	0.6	0.224
Vz	AFE	20.19	4.016	16.906	3.674	19.88	4.436
CLz	AFE	1.049	0.456	1.193	0.119	1.364	0.166
Cmax	AFE × h	1.41 × 10^8^	3.21 × 10^7^	1.51 × 10^8^	2.45 × 10^7^	1.32 × 10^8^	1.38 × 10^7^
C0	AFE × h	1.35 × 10^8^	6.63 × 10^7^	1.14 × 10^8^	5.13 × 10^7^	1.73 × 10^8^	8.36 × 10^7^

AFE is the abbreviation of average radiant efficiency units [p/s/cm^2^/sr]/[µW/cm^2^].

## Data Availability

Data are contained within the article and supplementary materials.

## Supplementary Materials

The following supporting information can be downloaded at: https://www.mdpi.com/article/10.3390/pharmaceutics16010075/s1, Figure S1: The emission wavelengths of siRNA LNPs with different formulations; Figure S2: The excitation wavelengths of siRNA LNPs with different formulations; Figure S3: The standard curve of TGFβ1 siRNA solution; Figure S4: Bands showing TGFβ1 protein expression in A549/T cells for the various siRNA LNPs; Figure S5: The proliferation inhibition rates of the various siRNA LNPs on A549/T cells; Figure S6: The fluorescence intensity-time curves of cypate-labeled siRNA LNPs in tumor; Figure S7: The changes in body weight of nude mice during the administration cycle; Figure S8: Immunohistochemical analysis of TGFβ1 protein expression in tumor tissue after administration; Figure S9: The pathological results for various organs of five groups after the efficacy experiment.
