# Supplementary material for: The Efficacy and Safety of Polyethylene Glycol Cholesterol- and Tocopherol Polyethylene Glycol 1000 Succinate-Modified Transforming Growth Factor β1 Small Interfering RNA Lipid Nanoparticles in the Treatment of Paclitaxel-Resistant Non-Small-Cell Lung Cancer
Source: Pharmaceutics. 2024 Jan 4;16(1):75. doi: 10.3390/pharmaceutics16010075 (PMC10820430; doi:10.3390/pharmaceutics16010075)
Supplement: Supplementary file 1 [file pharmaceutics-16-00075-s001.zip › pharmaceutics-2704666-supplementary.pdf]

Supplementary data

1. Supplementary detailed method: Detection method of the contents and encapsulation efficiencies of siRNA in TGFβ1 siRNA LNPs in detail.

(1) Determination of emission wavelength and excitation wavelength of siRNA LNPs

A reference solution of 100 ng/mL TGFβ1 siRNA solution and three sample solutions of siRNA LNPs were placed in a 96-well plate. A microplate reader was used to determine the emission and excitation wavelengths of siRNA LNPs. For the emission wavelength, the fixed excitation wavelength was 485 nm, and the scanning emission wavelength was 520-600 nm. The results show that the emission wavelength scanning curves of siRNA LNPs with different formulations are similar, with maximum absorption around 530 nm, as shown in Figure S1. At the same time, referring to the RiboGreen instructions, 530 nm was ultimately chosen as the emission wavelength.

For the excitation wavelength, the fixed emission wavelength was 530 nm, and the scanning excitation wavelength was 440-498 nm. The results show that the emission wavelength scanning curves of siRNA LNPs with different formulations are similar, with small fluorescence intensity around 485 nm and a relatively flat curve. When the excitation wavelength exceeded 495 nm, the fluorescence intensity increased sharply, as shown in Figure S2. Referring to the RiboGreen instructions, 485 nm was finally chosen as the excitation wavelength.

(2) Standard curve of TGFβ1 siRNA

From the 2 μg/mL reference solution of TGFβ1 siRNA, 0, 50, 100, 200, 300, 400, and 600 μL were respectively taken. Then, 980, 930, 880, 780, 680, 580, 380 μL of 1×Tris EDTA (TE) buffer were respectively added. Then, 20 μL of 1% emulsifier OP was added, followed by 1 mL of RiboGreen dilute solution (200 times dilution from original solution), such that the final volume was 2 mL, and the final concentrations were 0, 50, 100, 200, 300, 400, and 600 ng/mL, respectively. The samples were added to a 96-well plate, and the standard curve of TGFβ1 siRNA was determined using the microplate reader.

(3) Detection of the contents and encapsulation efficiencies of siRNA in TGFβ1 siRNA LNPs

siRNA LNP sample solution (0.2 mL) was combined with 1 mL of 1% emulsifier OP, shaken for 2 min, and allowed to stand for 10 min. The samples were then diluted to 10 mL with 1×TE buffer as siRNA LNP depolymerization solution. Then, 0.2 mL of siRNA LNP depolymerization solution was combined with 0.8 mL of TE buffer, 1 mL of RiboGreen dilution solution (200 times dilution) was then added, mixed well as siRNA LNP detection solution, and 0.2 mL was then taken out and placed in a 96-well plate, of which the relative fluorescence intensity was measured using a microplate reader. The siRNA contents in the siRNA LNP samples were calculated according to the standard curve regression equation.

siRNA LNP solution (0.2 mL) was taken out and combined with 0.3 mL of DEPC-treated water, placed in a 300 KD ultrafiltration centrifuge tube, and centrifuged 5000×g for 30 min. The upper tube was then placed into a 50 mL centrifuge tube with addition of 9 mL of 1×TE buffer and 1 mL of 1% emulsifier OP, shaken for 2 min, and allowed to stand for 30 min as siRNA depolymerization solution. Then, 0.2 mL of siRNA depolymerization solution was taken out and combined with 0.8 mL of 1×TE buffer and 1 mL of RiboGreen dilute solution, mixed well as siRNA LNP detection solution, and the fluorescence intensity was measured. The concentration of siRNA encapsulated in the carrier was calculated according to the standard curve equation. Encapsulation rate (%) = encapsulated siRNA content/total siRNA content×100%.

2. Supplementary detailed method: Inhibition of TGFβ1 mRNA expression in A549/T cell after siRNA LNP intervention

The mRNA expression in these A549/T cells was detected according to the common qPCR method, detailed steps was as follows: Total RNA was extracted using the conventional TRIzol method. The $OD_{260/280}$ method was used to determine the concentration and purity of RNA, and the $OD_{260/280}$ of the extracted RNA was between 1.8 and 2.1, which meets the purity requirements. Then, the primescript RT master mix reagent kit was used to prepare a reaction system according to the instructions for reverse transcription reaction. The reverse transcription reaction conditions were as follows: First, hold at 37°C for 15 min, then at 85°C for 5 s, and then place on ice for 5 min. The product underwent the next PCR reaction. The cDNA generated by reverse transcription was subjected to real-time fluorescence quantitative PCR reaction. A reaction system was prepared according to the instructions of the Realtime PCR Master Mix (SYBR Green) kit. The prepared reaction system was added to a 96-well plate for real-time fluorescence quantitative PCR reaction. The reaction conditions for real-time fluorescence quantitative PCR were 95°C for 30 s pre-denaturation; 95°C for 15 s; 60°C for 20 s; 72°C for 40 s, 40 cycles of reaction; 95°C for 15 s; 60°C for 60 s; 95°C for 15 s melting.

3. Supplementary figures

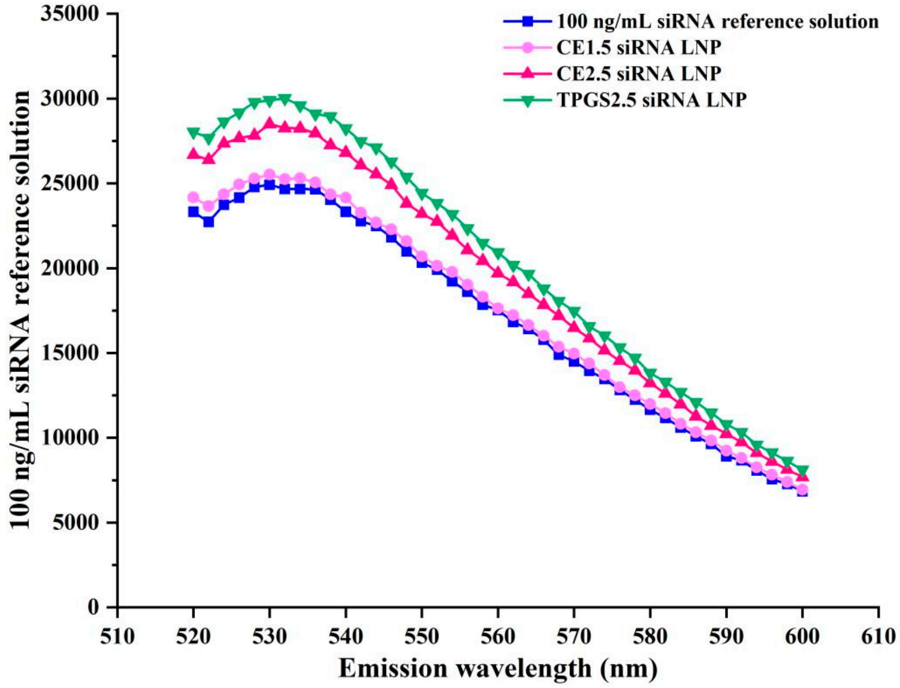

Figure S1. The emission wavelengths of siRNA LNPs with different formulations. The emission wavelength curves of the four solutions are similar.

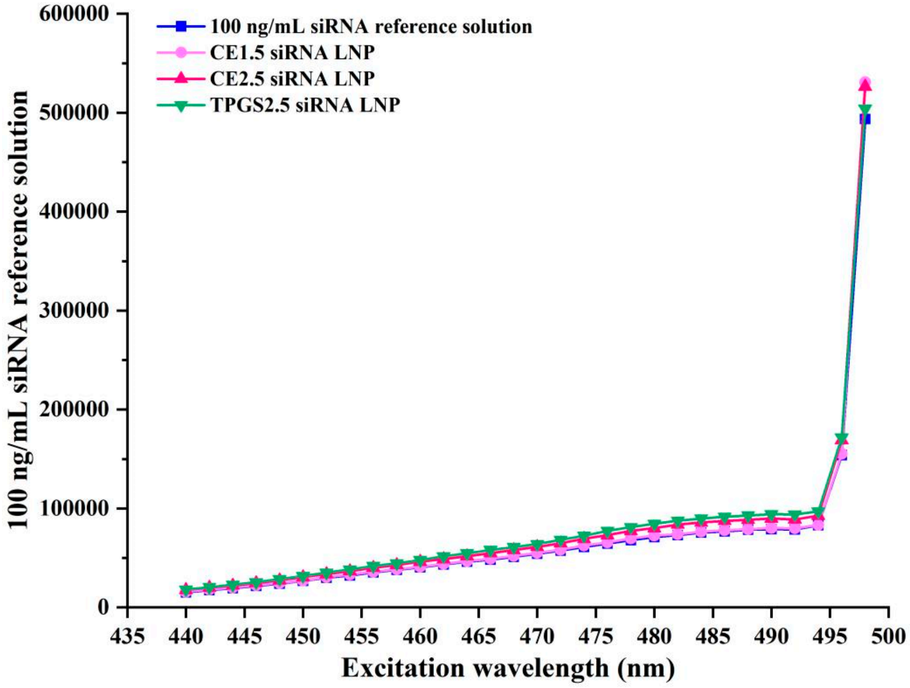

Figure S2. The excitation wavelengths of siRNA LNPs with different formulations. The excitation wavelength curves of the four solutions are similar.

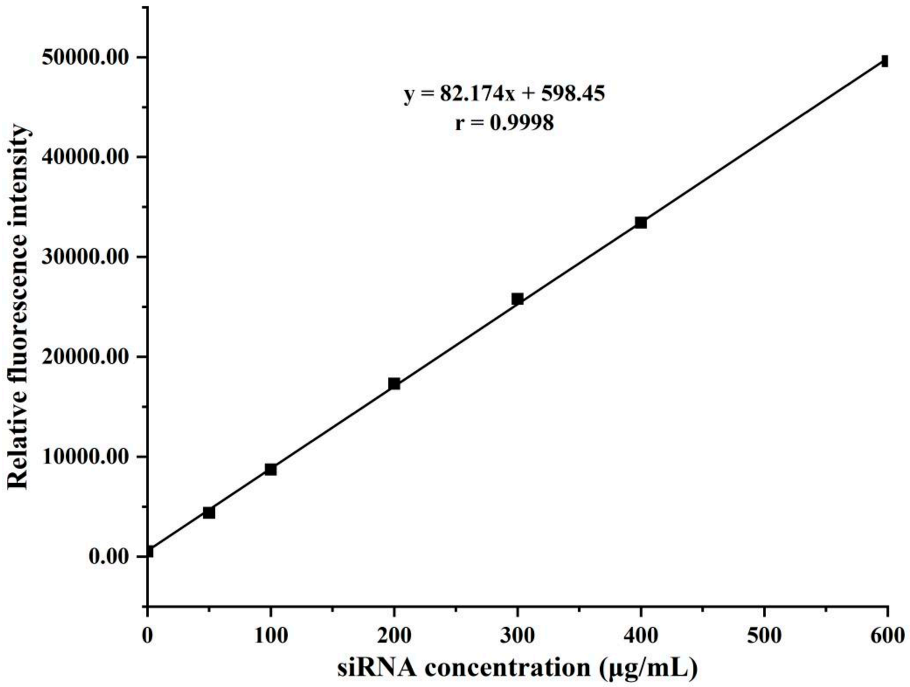

Figure S3. The standard curve of TGFβ1 siRNA solution. The relative fluorescence intensity of siRNA was linear in the range of 0-600 ng/ml, and the correlation coefficient was greater than 0.999.

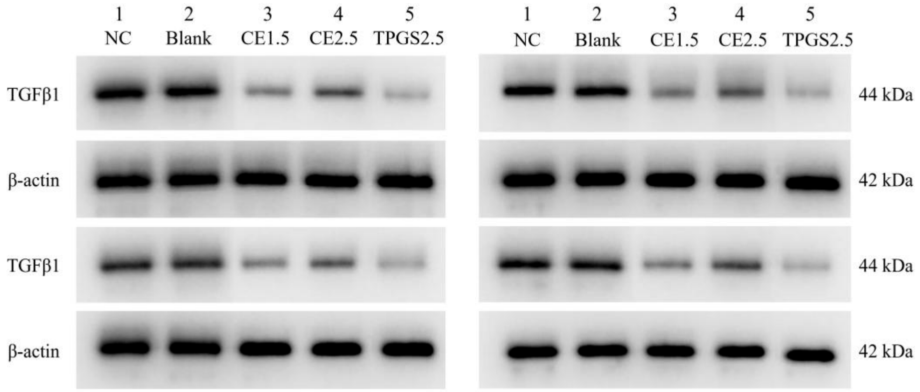

Figure S4. Bands showing TGFβ1 protein expression in A549/T cells for the various siRNA LNPs. 1. NC siRNA LNP; 2. Blank LNP; 3. CE1.5 TGFβ1 siRNA LNP; 4. CE2.5 TGFβ1 siRNA LNP; 5. TPGS2.5 TGFβ1 siRNA LNP. The grayscale values of the CE1.5, CE2.5, and TPGS2.5 LNP bands were lower than those of the NC LNP group and the blank LNP group.

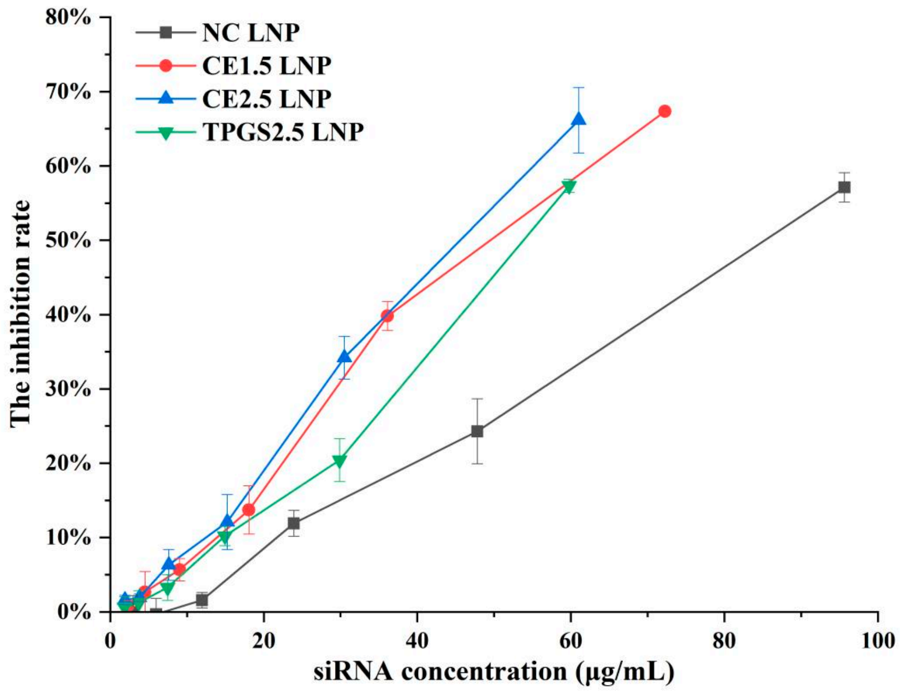

Figure S5. The proliferation inhibition rates of the various siRNA LNPs on A549/T cells (n=3). The cell inhibition curves of CE1.5, CE2.5, and TPGS2.5 LNP were similar.

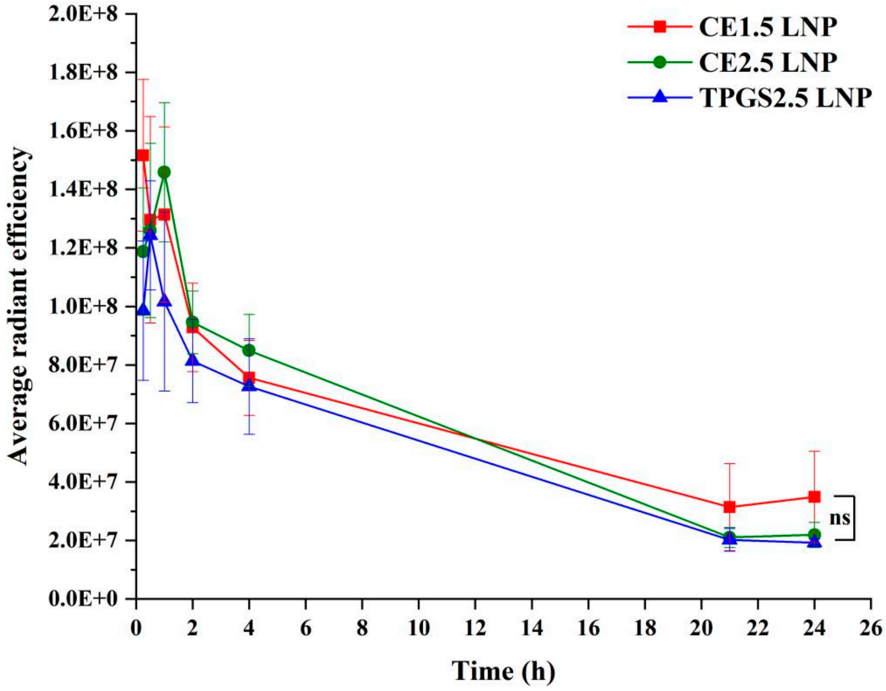

Figure S6. The fluorescence intensity-time curves of cypate-labeled siRNA LNPs in tumor (n=5. ns, *P*>0.05). The fluorescence intensity gradually decreases with time. The fluorescence intensity-time curves of CE1.5, CE2.5, and TPGS2.5 LNP were similar.

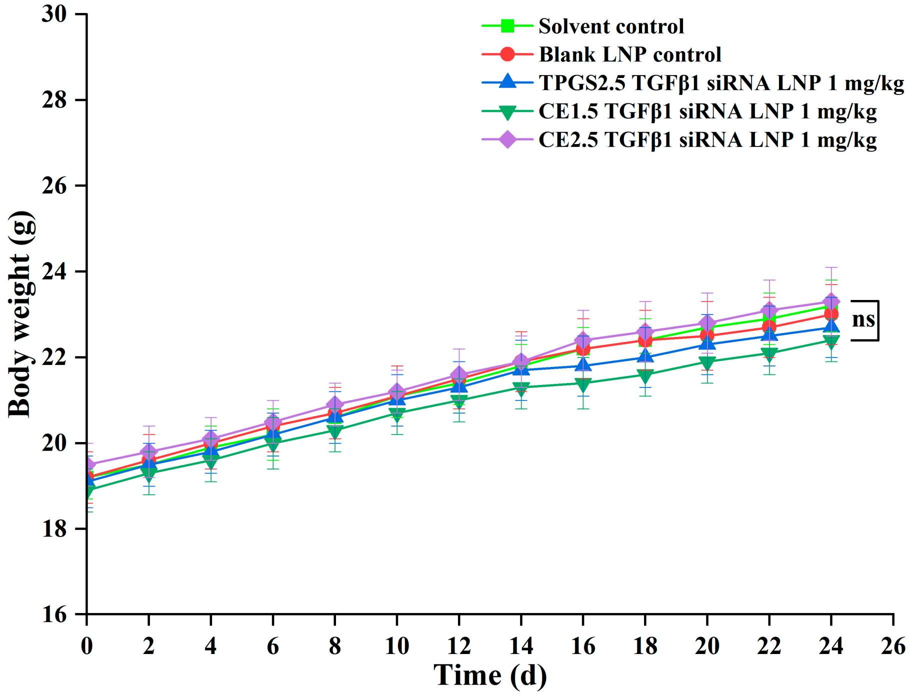

Figure S7. The changes in body weight of nude mice during the administration cycle (n=7. ns, *P*>0.05). The weight of all five groups of nude mice did not decrease.

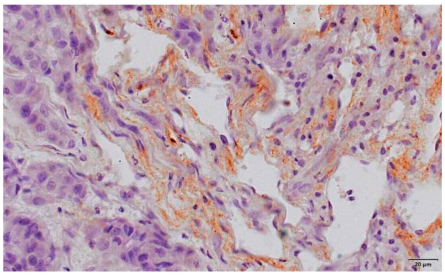

Solvent control

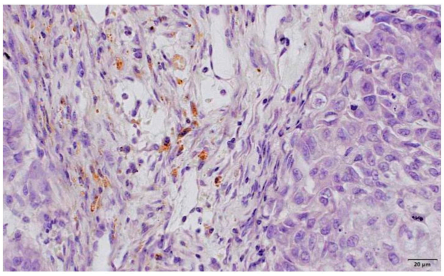

CE1.5 LNP

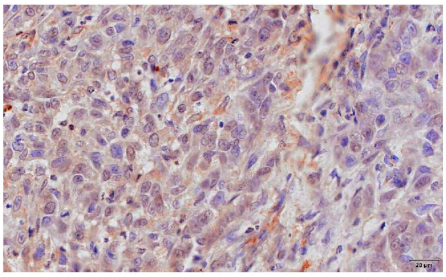

Blank LNP control

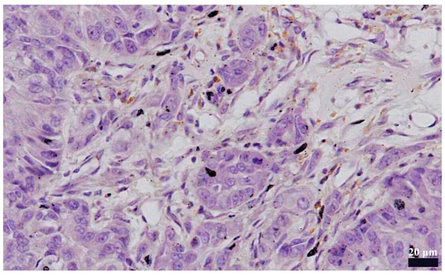

CE2.5 LNP

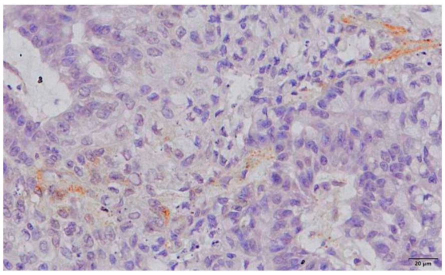

TPGS2.5 LNP

Figure S8. Immunohistochemical analysis of TGFβ1 protein expression in tumor tissue after administration (n=6). The optical density value of TGFβ1 staining in the CE1.5 LNP, CE2.5 LNP, and TPGS2.5 LNP groups was obvious reduced compared to the control groups.

4. Supplementary detailed results: The organ pathology detailed results after the *in vivo* treatment of paclitaxel-resistant lung adenocarcinoma

The pathological detailed results of various organs after the efficacy experiment are shown in Figure S9. The observations from pathological analyses are as follows:

(1) Tumor. Solvent group and blank LNP group: There is a small central necrotic area, with the necrotic area ranging from 1/4 of the total area, and inflammatory cells infiltrate the necrotic area. TPGS2.5 LNP group: There are obvious apoptotic cells. There is a central necrotic area, with a necrotic area ranging from 1/3 to 1/2 of the total area, and inflammatory cells infiltrate the necrotic area. The CE1.5 and CE2.5 LNP groups are similar to the TPGS2.5 LNP group, with a necrotic area greater than 1/2-2/3 of the total area.

(2) Heart. Solvent group: The boundary of myocardial cells is not clear. Inflammatory cell infiltration can be seen in the interstitial tissue of the heart. The results for the blank LNP, TPGS2.5 and CE1.5 LNP groups are similar to those of the solvent group. For the CE2.5 LNP group, significant interstitial congestion is exhibited in heart tissue, with visible infiltration of inflammatory cells.

(3) Liver. Inflammatory cells are scattered around the lobules of liver blood vessels and in the liver parenchyma in each group. TPGS2.5 LNP: Slight degeneration of liver cells and slight congestion of liver sinuses. CE1.5 LNP group: Hepatocellular degeneration, hepatic sinus congestion. CE2.5 LNP group: Hepatocellular degeneration, hepatic sinus congestion.

(4) Spleen: Normal in all groups.

(5) Lungs: All groups exhibited mild congestion of lung tissue, with occasional or infiltration of inflammatory cells in the pulmonary interstitium. Solvent group: No edema in lung tissue. The blank LNP group: No edema in lung tissue. Slight thickening of alveolar walls. The TPGS2.5, CE1.5, and CE2.5 LNP groups all exhibited slight thickening of alveolar walls and thickening of capillary walls.

(6) Kidney: Solvent group and blank LNP group: Small amount of local congestion in the glomerulus with infiltration of inflammatory cells. The other three groups all exhibited local congestion and infiltration of inflammatory cells. Occasional renal tubular swelling and interstitial edema were observed in the TPGS2.5 and CE1.5 LNP groups. The CE2.5 LNP group exhibited renal tubular swelling and renal interstitial edema.

(7) Stomach: Normal in the solvent group. Blank LNP group exhibited slight inflammatory cell infiltration. The TPGS2.5, CE1.5, and CE2.5 LNP groups all exhibited mild congestion in the gastric submucosa and a small amount of inflammatory cell infiltration.

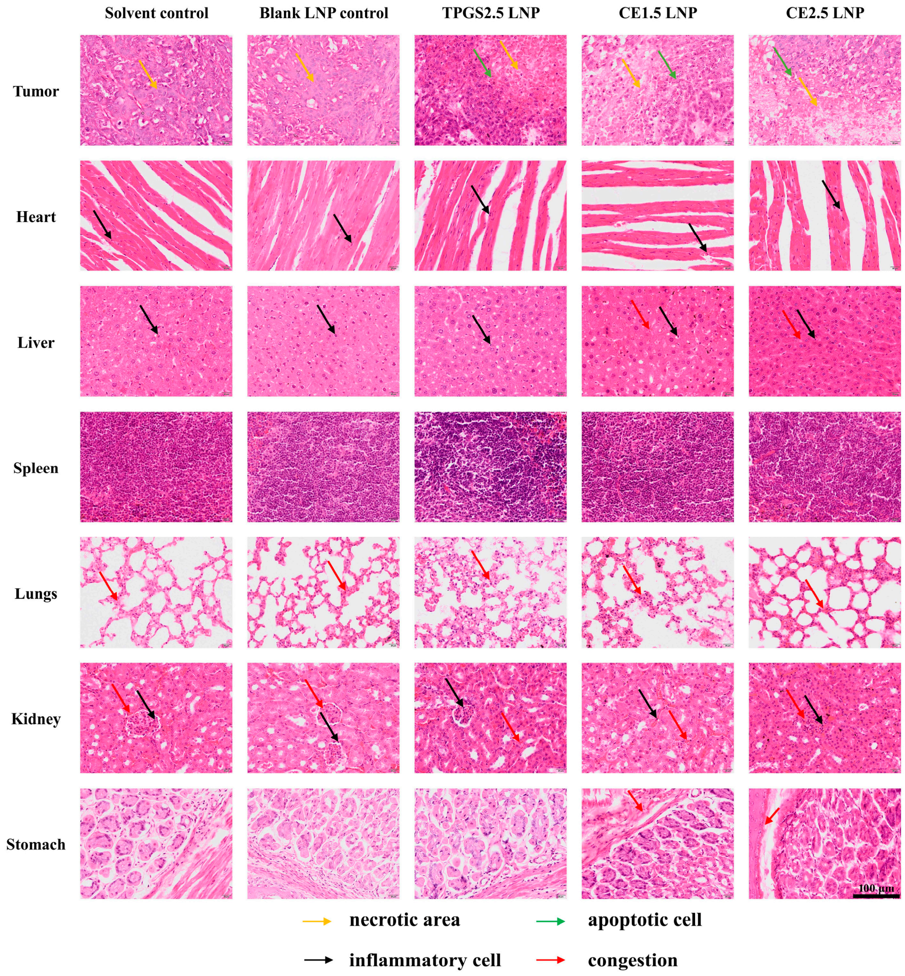

Figure S9. The pathological results for various organs of five groups after the efficacy experiment. The four types of LNP had a certain toxicity mainly related to inflammation on the lungs, liver, kidneys, and stomach.
